# MFG-E8 Released by Apoptotic Endothelial Cells Triggers Anti-Inflammatory Macrophage Reprogramming

**DOI:** 10.1371/journal.pone.0036368

**Published:** 2012-04-30

**Authors:** Marie-Joëlle Brissette, Stéphanie Lepage, Anne-Sophie Lamonde, Isabelle Sirois, Jessika Groleau, Louis-Philippe Laurin, Jean-François Cailhier

**Affiliations:** 1 Centre de Recherche du Centre hospitalier de l'Université de Montréal (CRCHUM), Université de Montréal, Montreal, Quebec, Canada; 2 Institut du Cancer de Montréal, Montreal, Quebec, Canada; 3 Nephrology Division, Centre hospitalier de l'Université de Montréal (CHUM), Department of Medicine, Université de Montréal, Montreal, Quebec, Canada; McGill University, Canada

## Abstract

Apoptotic endothelial cells are an important component of the “response to injury” process. Several atherosclerosis risk factors such as hyperglycemia and oxidized low-density lipoproteins, and immune injuries, such as antibodies and complement, induce endothelial cell apoptosis. While endothelial cell apoptosis is known to affect neighboring vascular wall cell biology, its consequences on macrophage reprogramming are ill defined. In this study, we report that apoptosis of human and mouse endothelial cells triggers the release of milk fat globule-epidermal growth factor 8 (MFG-E8) and reprograms macrophages into an anti-inflammatory cells. We demonstrated that MFG-E8 is released by apoptotic endothelial cells in a caspase-3-dependent manner. When macrophages were exposed to conditioned media from serum-starved apoptotic endothelial cells, they adopt a high anti-inflammatory, low pro-inflammatory cytokine/chemokine secreting phenotype that is lost if MFG-E8 is absent from the media. Macrophage treatment with recombinant MFG-E8 recapitulates the effect of conditioned media. Finally, we showed that MFG-E8-mediated reprogramming of macrophages occurs through increased phosphorylation of signal transducer and activator of transcription-3 (STAT-3). Taken together, our study suggests a key role of MFG-E8 release from apoptotic endothelial cells in macrophage reprogramming and demonstrates the importance of the apoptotic microenvironment in anti-inflammatory macrophage responses.

## Introduction

Apoptosis, an ubiquitous form of cell death, occurs during embryogenesis in normal tissues and during inflammation. It has been classically associated with a silent form of cell dismissal [1]. However, recent evidence suggests that apoptotic cells can modulate their microenvironment and neighboring cell biology. Apoptotic cells are known to release “eat-me” and “find-me” signals aimed at coordinating the non-phlogistic recruitment of professional phagocytes to allow swift clearance of apoptotic cells and inhibition of neutrophil influx [2], [3], [4], [5], [6], [7]. Mounting evidence indicates that the paracrine component of the apoptotic program is not limited to the regulation of leukocyte trafficking, but also prepares the cellular microenvironment for remodeling after apoptotic cell deletion. Apoptotic endothelial cells (EC) are a key component of the “response to injury” process. It is recognized that most atherosclerosis risk factors (such as hypertension [8], hyperglycemia [9] and oxidized low-density lipoproteins [10]) and antibody-complement-mediated immune injuries [11] induce EC apoptosis, which can generate a local microenvironment that will affect cell survival [12], [13], [14], [15], activation [16], [17] and differentiation [13], [18] of neighboring vascular wall cells. Apoptotic cells can modify their local environment through classical and non-classical secretion of various biological agents [15]. However, the reprogramming consequences of this apoptotic microenvironment on macrophages have not yet been fully characterized.

Macrophages are essential for initiating both inflammation and the repair of injured tissues [19]. In inflammation, they respond destructively to the damage identified; they also promote resolution of inflammation and contribute to tissue repair [20]. Initiation of inflammation, tissue damage and fibrosis are promoted by macrophages through reprogramming induced by the local microenvironment [11], [20], [21], [22], [23]. Indeed, the macrophage phenotype is affected by various signaling cues that vary according to the inflammation phase when they are recruited [20], [23]. The injury-inducing and repair-promoting role of macrophages has been well described: macrophage depletion during the fibrosis phase reduces scarring, but depletion during recovery inhibits matrix degradation [24]. Furthermore, there is evidence to suggest that macrophages have a crucial role in conditions where endothelial apoptosis is present [25], [26], [27], [28].

The local environment can affect macrophage phenotype and influence the nature of its inflammatory response. Phenotypes are dynamic; they can switch from pro-inflammatory to anti-inflammatory and *vice versa* [29]. Therefore, two macrophage phenotypes can be identified: pro-inflammatory (M1) and anti-inflammatory (M2). Pro-inflammatory M1 macrophages stem from classical and innate activation. They produce pro-inflammatory cytokines such as tumor necrosis factor, monocyte chemotactic protein-1 (MCP-1) and macrophage inflammatory protein-2 (MIP-2). Anti-inflammatory, pro-repair M2 macrophages derive from alternative activation or reprogramming induced by the phagocytosis of apoptotic cells. This phenotype is characterized by the production of anti-inflammatory molecules such as interleukin-10 (IL-10), transforming growth factor (TGF)-β_1_, and vascular endothelial growth factor (VEGF) [30], [31], [32]. However, considering phenotype as described is a simplistic view, as these macrophage phenotypes represent a continuum where transitional states are possible [33].

Our previous work, via a proteomic approach, suggested that milk fat globule-epidermal growth factor 8 (MFG-E8) could be secreted by apoptotic EC [15]. It could be produced by various cell types and, essentially, by activated macrophages. MFG-E8 is crucial for the phagocytosis of apoptotic cells [34] and in macrophage activation [35], [36]. We have further investigated MFG-E8 release by apoptotic endothelial cells due to its importance in inflammation and especially in macrophage function.

Here, we show MFG-E8 release by apoptotic EC. We propose that apoptotic EC may be the initial cellular source of MFG-E8, before its production by activated macrophages [34]. Our study suggests that apoptotic cell-conditioned media contribute to macrophage reprogramming into anti-inflammatory, pro-repair macrophages, via MFG-E8 release in a phagocytosis-independent manner.

## Results

### Apoptotic endothelial cell-conditioned medium contains MFG-E8 and could program macrophages

**Figure 1:**
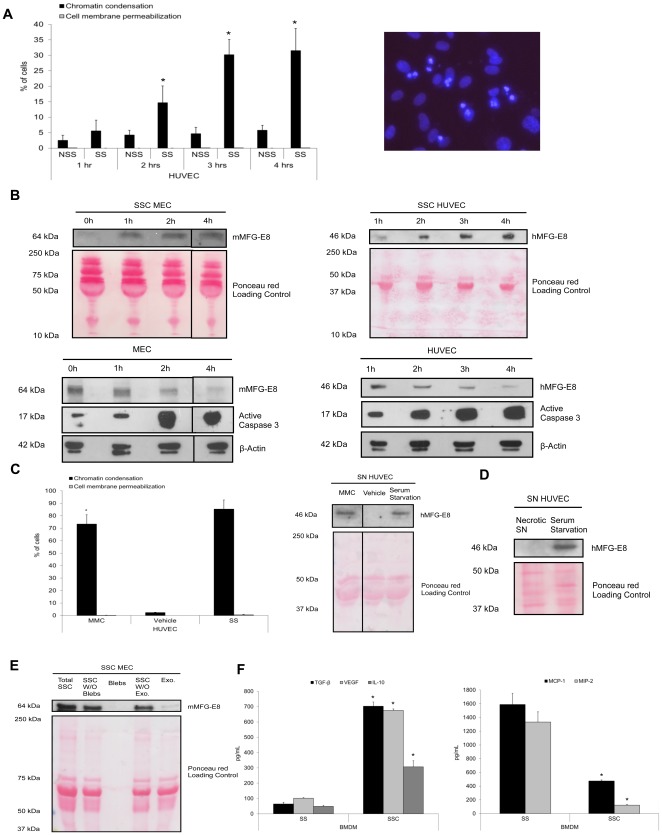
Apoptotic EC-conditioned media contain MFG-E8 and reprogram macrophages. **A** Percentage of cells with increased chromatin condensation and cell membrane permeabilization (as evaluated with HO and PI staining) in HUVEC exposed to normal medium with growth factors without serum (normal serum-starved, NSS) or serum starvation (SS) for 1 h to 4 h (*p<0.001 versus Normal, *n* = 3). Example of HO/PI staining on serum starved HUVEC for 4 h showing chromatin condensation, right panel. **B** MEC and HUVEC were serum-starved for 1 h to 4 h. Supernatants (upper panels) and cells (lower panels) were harvested. Immunoblotting of MEC protein extracts showed that MFG-E8 levels decreased over time in parallel with increased active caspase-3 levels (lower left panel). HUVEC also exhibited reduced intracellular MFG-E8 levels over time (lower right panel). MFG-E8 levels increased over time in serum-starved conditioned medium (SSC) from EC (upper panels). β-Actin and Ponceau red staining were loading controls. Representative of 3 experiments. **C** Percentage of cells with increased chromatin condensation and cell membrane permeabilization (as evaluated with HO and PI staining) in HUVEC exposed to MMC 0.01 mg/mL or vehicle in normal medium and serum starvation (as positive control) for 15 h (left panel), *p<0.0001 versus vehicle, *n* = 3. Immunoblot for hMFG-E8 in supernatant of EC treated with MMC (right panel). Ponceau red staining is shown as loading control. Representative of 2 experiments. **D** Immunoblot for hMFG-E8 in supernatants conditioned by necrotic HUVEC (3 freeze-thaw cycles) and serum-starved HUVEC as positive control. Ponceau red staining included as loading control. Representative of 2 experiments. **E** Immunoblot for mMFG-E8 from total medium conditioned by apoptotic EC (Total SSC), supernatant after removal of apoptotic blebs by centrifugation at 50 000 g (SSC without (W/O) blebs) and apoptotic blebs (Blebs) purified from total SSC by centrifugation, supernatant obtained from the supernatant after 50 000 g and 200 000 g centrifugation (SSC W/O exo.) and exosome-like nanovesicle fraction pelleted after the 200 000 g centrifugation (Exo.). Proteins from equal initial volumes were precipitated by TCA. Ponceau red staining is shown as loading control of samples. Representative of 2 experiments. **F** MEC were serum-starved for 4 h, the SSC were harvested, centrifuged to remove apoptotic cells. Murine macrophages were exposed to SSC or serum starvation (SS) for 24 h. ELISA were performed for TGF-β_1_, VEGF, IL-10, (left panel) MCP-1 and MIP-2 (right panel), *p<0.05, representative of *n* = 14, 12, 4, 7 and 9 separate experiments respectively.

We first assessed whether apoptotic EC could release MFG-E8. Apoptosis was induced *in vitro* by serum starvation (SS) for 4 h as reported previously [12], [13], [15], [37], [38]. This model is relevant to situations where EC apoptosis is found: chronic transplant vasculopathy and ischemia-reperfusion [11], [39]. In our study, serum-starved human umbilical vein endothelial cells (HUVEC) (Figure 1a), evaluated with Hoechst 33342 (HO) and propidium iodide (PI) staining, showed a progressive time-dependent increase of chromatin condensation in the absence of cell membrane permeabilization, indicative of apoptosis, as described elsewhere [12], [13], [15], [37], [38]. Necrosis, indicated by cell membrane permeabilization (inclusion of PI), was not significantly induced by SS. Chromatin condensation was present after 2 h of SS (Figure 1a), concomitantly to caspase-3 activation (Figure 1b, lower panels). Murine EC (MEC) and HUVEC apoptosis was associated with MFG-E8 release in the serum starved-conditioned media (SSC) (Figure 1b, upper panels), whereas the intracellular MFG-E8 content declined in EC, whilst simultaneously exhibiting heightened expression of active caspase-3 fragments (Figure 1b, lower panels). No significant differences were found in MFG-E8 release between BALB/c and C57BL/6 serum-starved EC (data not shown). We used mitomycin C (MMC) as another pro-apoptotic stimulus [15], [37]. MMC treatment of EC augmented the percentage of cells with chromatin condensation and promoted MFG-E8 release (Figure 1c). Furthermore, MFG-E8 was absent from media conditioned by necrotic EC suggesting that this protein is not released passively as a consequence of cell membrane permeabilization (Figure 1d). Since MFG-E8 can be secreted as a soluble or as a small membrane vesicle protein (like exosomes), we then investigated which form serum-starved EC released MFG-E8. Equal volumes of total unfractionated SSC were centrifuged at 50 000 g to remove apoptotic bodies and apoptotic cells. Supernatants and bleb pellets were collected. Obtained supernatants were then ultracentrifuged at 200 000 g to sediment small membrane vesicles, the resulting supernatants and vesicle pellets were harvested. MFG-E8 levels were detected in total unfractionated SSC, in supernatants from the centrifugation at 50 000 g and in supernatants from the 200 000 g ultracentrifugation (Figure 1e). The small membrane vesicle fraction contained MFG-E8, but at lower levels than in the supernatants. MFG-E8 was absent in the apoptotic blebs fraction (Figure 1e). These results suggest that MFG-E8 is mostly released by apoptotic EC as a soluble molecule rather than associated to small membrane vesicles.

To evaluate the phenotypic consequences of SSC on macrophage reprogramming, murine bone marrow-derived macrophages (BMDM) were stimulated with SSC for 24 h. Experiments performed with BMDM from C57BL/6 and BALB/c mice showed no differences in cytokine production between strains (data not shown). They produced more TGF-β_1_, VEGF and IL-10 in response to SSC from apoptotic EC than control serum-starved macrophages (Figure 1f, left panel, values in Table 1). Furthermore, the production of pro-inflammatory chemokines MCP-1 and MIP-2 was significantly lower with SSC than with SS exposure (Figure 1f, right panel, values in Table 1). Similar results were obtained with human monocytes-derived macrophages (HMDM). TGF-β_1_ production by HMDM was increased 2.5 times in response to SSC from apoptotic EC compared to serum starvation alone whereas production of pro-inflammatory cytokines IL-8, MCP-1 and IL-6 were 90%, 93% and 97% reduced respectively with SSC exposure compared to SS (*p*<0.001, n = 3). Therefore, macrophages exposed to conditioned media from serum-starved apoptotic endothelial cells, adopt a high anti-inflammatory, low pro-inflammatory cytokine/chemokine secreting phenotype.

**Table 1:** Phenotypic analysis of murine bone-marrow-derived macrophages exposed to SSC vs SS.

	TGF-β	VEGF	IL-10	MIP-2	MCP-1
SSC	703.2±27.65	675.74±8.95	307.15±39.07	121.72±14.77	474.20±14.77
SS	64.29±10.99	101.25±4.69	49.23±5.83	1332.96±148.46	1589.45±160.07

Data are presented as value mean ± SD in pg/mL; SSC: apoptotic serum-starved conditioned medium; SS: serum-starved medium; TGF: transforming growth factor; VEGF: vascular endothelial growth factor; IL: interleukin; MIP: macrophage inflammatory protein; MCP: monocyte chemotactic protein.

### Caspase-3 activation is necessary for MFG-E8 release and subsequent macrophage programming

**Figure 2:**
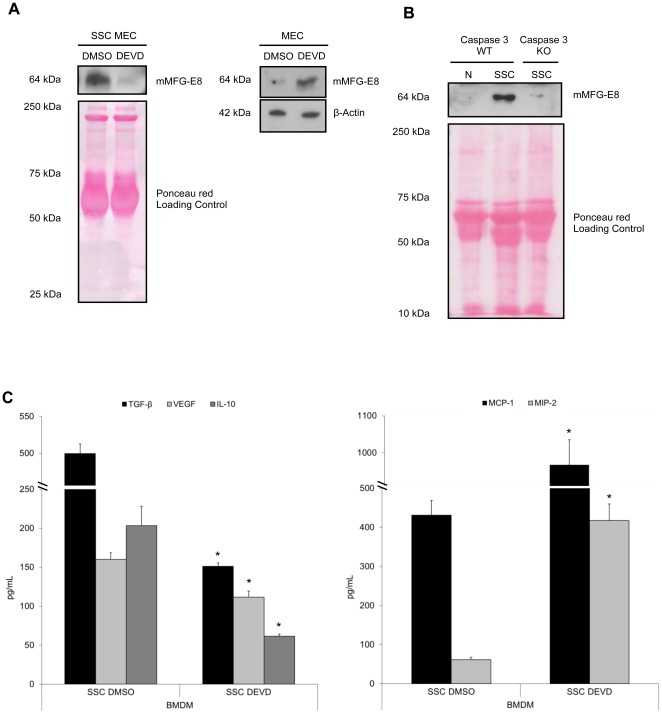
Caspase-3 activation is necessary for MFG-E8 release and subsequent macrophage reprogramming. MEC were pre-treated with an irreversible caspase-3 inhibitor, DEVD-FMK (SSC-DEVD, 100 µM) to prevent apoptosis, and then serum-starved for 4 h. Control MEC were pre-treated with vehicle (DMSO) for 2 h, washed and serum-starved for 4 h **A** Murine MFG-E8 was immunoblotted in SSC and cell extracts. DEVD-FMK-treated murine EC released less MFG-E8 compared to the vehicle (DMSO) (left panel), whereas their intra-cellular content remained higher than DMSO-treated EC (right panel). Ponceau red and β-Actin were loading controls. Representative of 3 experiments. **B** Immunoblot for murine MFG-E8 of SSC from caspase-3 KO EC compared to EC from WT mice. Representative of 2 experiments. **C** Murine macrophages produced more TGF-β_1_, VEGF, IL-10 (left panel) and less pro-inflammatory chemokines MCP-1 and MIP-2 (right panel) when exposed to media where apoptosis was not inhibited. *p<0.05, representative of *n* = 11, 9, 3, 5 and 8 separate experiments respectively.

Employing pre-treatment with an irreversible caspase-3 specific inhibitor, DEVD (SSC-DEVD), to inhibit EC apoptosis before SS, we investigated whether MFG-E8 liberation was dependent on caspase-3 activation. As illustrated in Figure 2a (left panel), MFG-E8 release from serum-starved MEC was greatly reduced after caspase-3 inhibition using DEVD compared to control (dimethylsulfoxide (DMSO)) pre-treated serum-starved MEC (SSC-DMSO). As expected, intracellular MFG-E8 levels were higher in DEVD than in DMSO-treated MEC (Figure 2a right panel). Furthermore, serum starvation of MEC from caspase-3 knockout (KO) mice was associated with the absence of MFG-E8 release in comparison to MEC from wild type (WT) mice (Figure 2b). Moreover, MEC apoptosis plays a role in macrophage reprogramming as the anti-inflammatory phenotype of macrophages was inhibited when exposed to serum-starved MEC treated with the caspase-3 inhibitor. As depicted in Figure 2c (left panel), the production of TGF-β_1_, VEGF and IL-10 was significantly decreased in BMDM subjected to SSC-DEVD compared to SSC-DMSO (Figure 2c, left panel, values in Table 2). However, we observed increased MCP-1 and MIP-2 production by SSC-DEVD-treated BMDM versus the control (Figure 2c, right panel, values in Table 2). Similar results were obtained with HMDM. IL-8, MCP-1 and IL-6 production were 1.5, 2 and 10 times increased respectively in response to SSC-DEVD compared to SSC-DMSO (*p*<0.001, n = 3). These results suggest that caspase-3-dependent MFG-E8 production from apoptotic EC programs macrophages into an anti-inflammatory phenotype.

**Table 2:** Caspase-3 dependent production of MFG-E8 in EC reprograms bone-marrow-derived macrophages.

	TGF-β	VEGF	IL-10	MIP-2	MCP-1
SSC-DEVD	151.28±4.17	111.49±7.89	61.64±2.69	417.33±42.48	966.94±67.87
SSC-DMSO	499.45±13.11	160.06±8.60	203.25±24.90	61.61±5.86	431.43±37.06

Data are presented as value mean ± SD in pg/mL; SSC: apoptotic serum-starved conditioned medium; TGF: transforming growth factor; VEGF: vascular endothelial growth factor; IL: interleukin; MIP: macrophage inflammatory protein; MCP: monocyte chemotactic protein.

### MFG-E8 plays an important and sufficient role in macrophage programming

**Figure 3:**
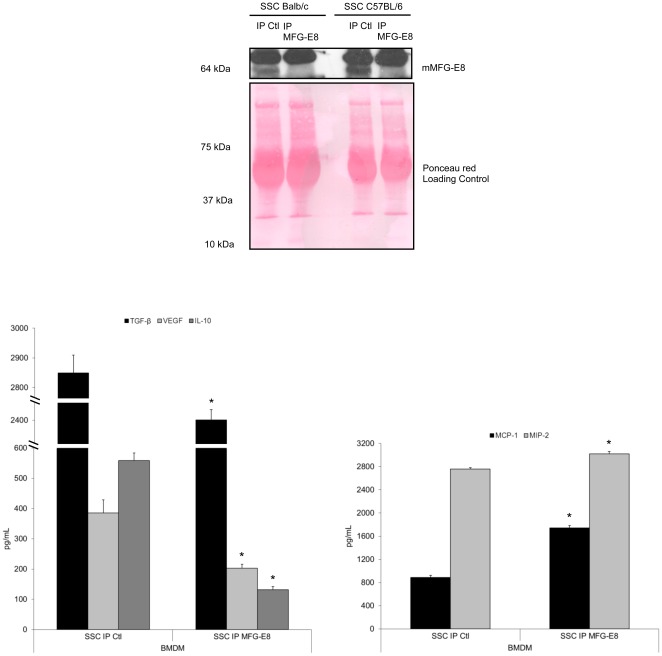
MFG-E8 immunoprecipitation from SSC alters macrophage reprogramming. Serum-Starved Conditioned medium (SSC) from MEC were treated with an anti-MFG-E8 antibody (or isotype control) to deplete the MFG-E8 content. Immunoblotting of MFG-E8 protein in SSC is shown for MEC (top panel). BMDM treated with SSC depleted of MFG-E8 produced less TGF-β_1_, VEGF, IL-10 (lower left panel), and more MCP-1 and MIP-2 (lower right panel). *p<0.05, representative of *n* = 2, 2, 3, 5 and 3 separate experiments respectively.

To clearly establish that macrophage programming in response to apoptotic MEC is dependent on MFG-E8 release from apoptotic MEC, we immunoprecipitated MFG-E8 from apoptotic MEC-conditioned media. Figure 3 demonstrates that the absence of MFG-E8 significantly inhibited macrophage reprogramming by apoptotic EC. Indeed, MFG-E8-immunodepleted SSC (Figure 3, upper panel) attenuated the production of the anti-inflammatory cytokines TGF-β_1_, VEGF and IL-10 (Figure 3, lower left panel, values in Table 3) and increased the production of the pro-inflammatory chemokines MCP-1 and MIP-2 (Figure 3, lower right panel, values in Table 3) compared to control.

**Table 3:** Immunoprecipitation of MFG-E8 in SSC reduces the anti-inflammatory reprogramming of macrophages by SSC.

	TGF-β	VEGF	IL-10	MIP-2	MCP-1
SSC IP-MFGE8	2401.33±34.79	202.78±13.12	131.91±10.40	3021.53±38.68	1743.41±41.15
SSC IP-ctl	2849.18±60.47	385.23±43.70	558.26±25.46	2757.97±23.73	887.54±38.36

Data are presented as value mean ± SD in pg/mL; SSC: apoptotic serum-starved conditioned medium; TGF: transforming growth factor; VEGF: vascular endothelial growth factor; IL: interleukin; MIP: macrophage inflammatory protein; MCP: monocyte chemotactic protein.

**Figure 4:**
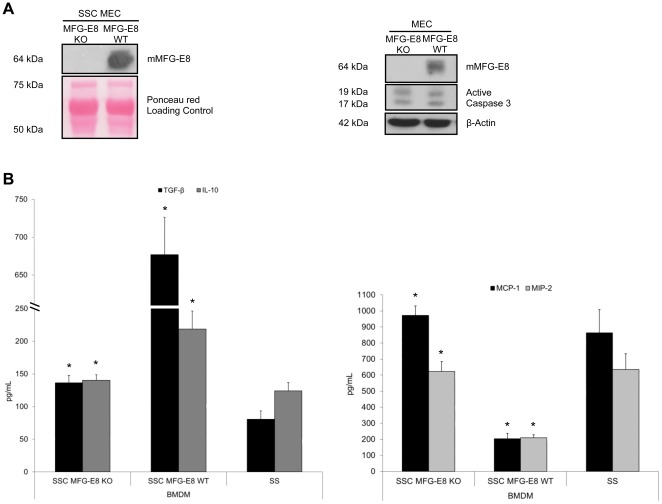
SSC from MFG-E8 KO mice do not reprogram macrophages into anti-inflammatory macrophages. **A** MFG-E8 KO or WT MEC were serum-starved for 4 h. Supernatants and cell extracts were immunoblotted for mMFG-E8 confirming KO status. Caspase-3 activation was similar between the 2 groups. **B** Murine macrophages were stimulated with Serum-Starved Conditioned medium (SSC) from MFG-E8 KO or WT EC. Supernatant were analyzed by ELISA. The results indicate that MFG-E8 in SSC is necessary to induce an anti-inflammatory macrophage phenotype. *p<0.05, representative of *n* = 5 separate experiments.

To further highlight the importance of EC-derived MFG-E8 in macrophage programming, we studied MEC derived from MFG-E8 KO mice. Immunoblotting for MFG-E8 content in supernatants and cell extracts from serum-starved KO and WT EC confirmed the absence of MFG-E8 in the KO (Figure 4a). In addition, immunoblotting revealed similar active caspase-3 levels in both KO and WT MEC (Figure 4a, right panel). SSC from MFG-E8 KO mice attenuated the production of anti-inflammatory cytokines TGF-β_1_ and IL-10 and increased the pro-inflammatory chemokines, MCP-1 and MIP-2, compared to SSC from MFG-E8 WT mice (Figure 4b, values in Table 4). SSC MFG-E8 KO-induced cytokine/chemokine production by macrophages was similar to that observed with the SS control (Figure 4b, values in Table 4). This suggests that the absence of MFG-E8 significantly altered macrophage programming by inducing comparable cytokine/chemokine production as seen in SS-stimulated macrophages.

**Table 4:** SSC from MFG-E8 KO mice reduces the anti-inflammatory reprogramming of macrophages.

	TGF-β	IL-10	MIP-2	MCP-1
SSC MFG-E8 KO	136.56±11.06	140.11±8.61	622.70±62.00	972.82±58.59
SSC MFG-E8 WT	676.93±49.23	218.72±27.32	209.42±19.16	203.65±33.51
SS	80.80±12.49	124.01±12.90	635.39±97.45	863.23±145.10

Data are presented as value mean ± SD in pg/mL; SSC: apoptotic serum-starved conditioned medium; TGF: transforming growth factor; IL: interleukin; MIP: macrophage inflammatory protein; MCP: monocyte chemotactic protein.

**Figure 5:**
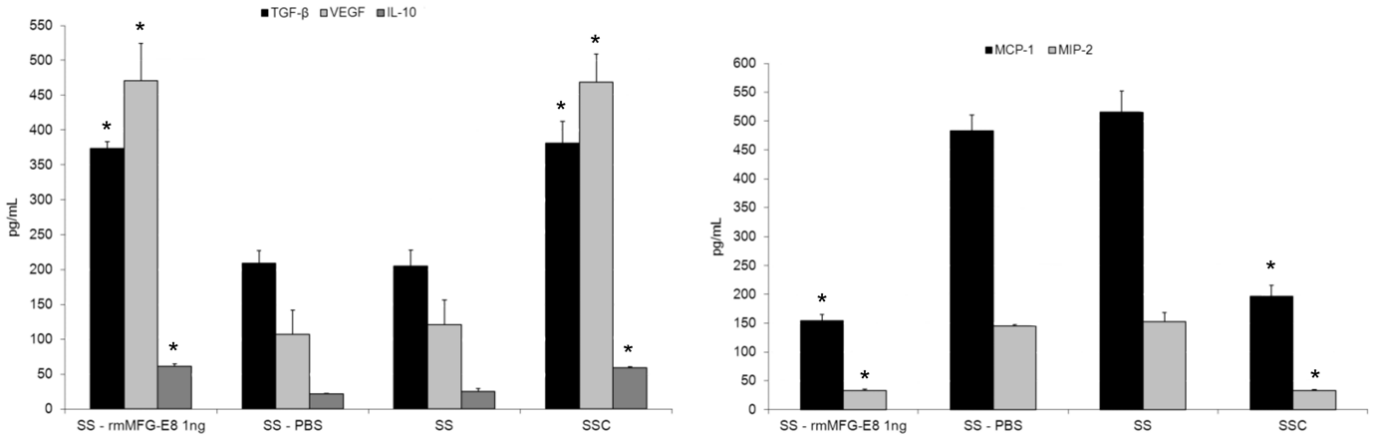
Recombinant murine MFG-E8 recapitulates SSC-induced macrophage reprogramming. Murine macrophages were stimulated with rmMFG-E8 (1 ng/mL) resuspended in RPMI (SS), vehicle (PBS), SS or SSC for 48 h and supernatant were harvested. rmMFG-E8 induced an anti-inflammatory macrophage phenotype with an increased production of TGF-β_1_, VEGF and IL-10 and reduced MCP-1 and MIP-2 compared to the vehicle control (PBS resuspended in SS). *p<0.05 vs respective controls, mean ± SD , representative of *n* = 3 separate experiments.

To demonstrate the essential role of MFG-E8 in reprogramming macrophage phenotype, we performed studies with recombinant murine (rm)MFG-E8 at the same concentration as found in SSC, 1 ng/ml (data not reported). Macrophages were stimulated with rmMFG-E8 (1 ng/mL), phosphate buffered saline (PBS) (vehicle for rmMFG-E8), SS or SSC for 48 h and the supernatants were harvested. Stimulation of macrophages with rmMFG-E8 increased production of the anti-inflammatory cytokines TGF-β_1_, VEGF and IL-10 and decreased the production of pro-inflammatory chemokines MCP-1 and MIP-2 compared to control PBS-treated macrophages (Figure 5, values in Table 5). Chemokine/cytokine production by macrophages treated with rmMFG-E8 was similar to that seen with SSC-treated macrophages. Similar results were obtained with HMDM. TGF-β_1_ production by HMDM was increased by 46% in response to rhMFG-E8, whereas production of pro-inflammatory cytokines IL-8 and MCP-1 were reduced by 73% and 70% respectively with rhMFG-E8 treatment compared to control PBS-treated macrophages (*p*<0.001, n = 2). Taken together, these results demonstrated the importance of MFG-E8 in the induction of an anti-inflammatory macrophage phenotype.

**Table 5:** rmMFG-E8 reproduces the anti-inflammatory macrophage phenotype.

	TGF-β	VEGF	IL-10	MIP-2	MCP-1
SS+ rmMFG-E8	373.16±10.02	470.82±53.29	61.14±3.69	32.65±3.02	154.69±10.61
SS+PBS	209.27±18.04	107.35±34.55	21.94±0.70	145.13±2.75	483.50±27.13

Data are presented as value mean ± SD in pg/mL; TGF: transforming growth factor; VEGF: vascular endothelial growth factor; IL: interleukin; MIP: macrophage inflammatory protein; MCP: monocyte chemotactic protein.

### Apoptotic endothelial cell-conditioned media activated the signal transducer and activator of transcription-3 (STAT-3) pathway in macrophages

**Figure 6:**
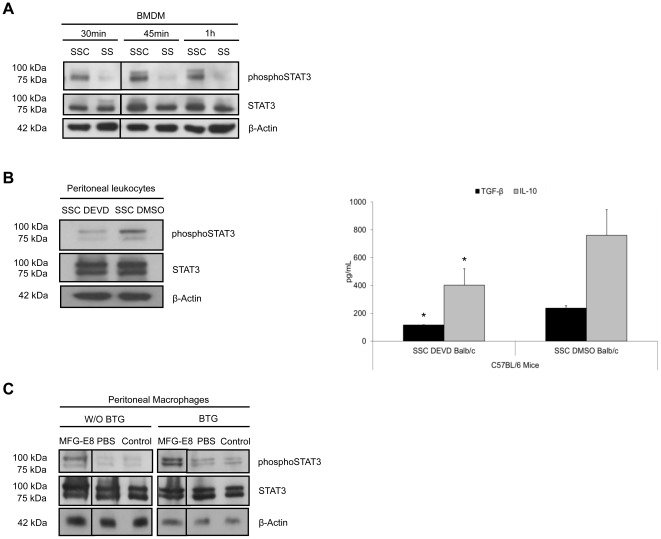
STAT-3 activation in macrophage reprogramming by apoptosis-conditioned media. **A** BMDM were stimulated with Serum-Starved Conditioned medium (SSC) or serum starvation (SS) for 30 minutes to 1 h. Total protein extracts were harvested and immunoblotted for phospho-STAT-3, STAT-3 and β-actin as loading control. Phosphorylated STAT-3 levels are higher after SSC stimulation compared to SS. Representative of 4 experiments. **B** C57BL/6 mice were pre-conditioned with SSC-DEVD or SSC-DMSO intraperitoneally for 3 h. Experimental peritonitis was induced with thioglycollate for 2 h and followed by peritoneal lavage to harvest peritoneal cellular exudates and supernatants. Protein extracts from the cellular exudates showed increased STAT-3 phosphorylation in mice pre-conditioned with SSC-DMSO compared to SSC-DEVD (**B**, left panel). ELISA of the supernatants revealed that SSC-DMSO pre-treatment increased TGF-β_1_ and IL-10 production compared to SSC-DEVD (**B**, right panel). *p<0.05, representative of *n* = 3 separate experiments. **C** MFG-E8 pre-conditioning increased STAT-3 phosphorylation compared to PBS pre-conditioned or control immunomagnetically-isolated peritoneal macrophages prior to Brewer thioglycollate (BTG) administration (W/O BTG). STAT-3 activation persisted and increased further 2 h following the induction of BTG peritonitis (BTG) in pre-conditioned macrophages. Total STAT-3 levels are depicted. β-Actin were loading controls. Representative of 2 experiments.

STAT family of transcription factors are involved in reprogramming of macrophages. STAT-1 activation is classically associated with the pro-inflammatory cytotoxic macrophage phenotype, whereas STAT-3 activation characterizes the pro-repair macrophages [20]. We, therefore, studied STAT-3 activation in macrophage reprogramming by apoptosis-conditioned media. Phosphorylated STAT-3 levels were higher after SSC stimulation compared to SS in BMDM (Figure 6a). We tested an experimental peritonitis model to assess the *vivo* reprogramming of macrophages by SSC. Pre-conditioning of peritoneal leukocytes with SSC-DMSO for 3 h increased STAT-3 phosphorylation in the cellular extracts 2 h after the induction of Brewer thioglycollate (BTG) peritonitis in mice compared to SSC-DEVD (Figure 6b, left panel). This, in turn, resulted in increased production of TGF-β_1_ and IL-10 (Figure 6b, right panel, see Table 6 for values). In additional studies, we determined the role of rmMFG-E8 (0.6 µg) in resident peritoneal macrophage pre-conditioning in STAT-3 activation. The administration of rmMFG-E8 increased the levels of STAT-3 phosphorylation in immunomagnetically-isolated peritoneal macrophages compared to PBS and unmanipulated control, prior to BTG injection. This STAT-3 activation persisted and increased further after BTG-induced peritonitis in isolated peritoneal macrophages compared to both controls (Figure 6c), indicating that MFG-E8 activated the STAT-3 pathway. Altogether, these results suggest that STAT-3 activation is present in the observed anti-inflammatory reprogramming of macrophages.

**Table 6:** Apoptosis-conditioned media *in vivo* pre-conditioning reprogram peritoneal macrophages.

	TGF-β	IL-10
SSC-DEVD	116.54±1.21	401.88±108.47
SSC-DMSO	237.55±17.64	760.50±184.76

Data are presented as value mean ± SD in pg/mL; SSC: apoptotic serum-starved conditioned medium; TGF: transforming growth factor; IL: interleukin.

## Discussion

Apoptotic cells release various elements that modify their microenvironment. This includes numerous chemokines or chemokine-like compounds, such as lysophosphatidylcholine [3], fractalkine [40] and nucleotides [41]. Existing evidence indicates that apoptotic EC induces resistance to apoptosis and contributes to changes in the phenotype of neighboring vascular wall cells [12], [13]. EC apoptosis, through cathepsin L release, degrades perlecan and generates the pro-fibrotic fragment LG3 [37]. Recently, other reports have suggested that the apoptotic milieu could also promote survival [14] and increase the phagocytosis of apoptotic cells by macrophages [42].

Apoptotic cells could activate classical and non-classical secretion pathways involving the exosomal release of proteins. Using proteomic analysis of media conditioned by apoptotic cells, we have previously suggested that MFG-E8 could be secreted, perhaps from the exosomal compartment [15]. However, this observation warranted further evaluation as presented here. Dendritic cells can secrete MFG-E8 through the release of exosomes [43]. Macrophages produce MFG-E8 upon activation whereas resident macrophages do not [34].

Our data highlights caspase-3-dependent MFG-E8 release by apoptotic EC as the primary source of an important protein introducing a novel mechanism of macrophage programming by the microenvironment. This apoptosis-conditioned microenvironment induces a phenotypic switch in macrophages, promoting anti-inflammatory and pro-repair macrophages, independent of apoptotic cell phagocytosis-induced reprogramming of macrophages. It suggests that, in addition to the anti-inflammatory function of apoptotic cells *per se* through their engulfment by macrophages and subsequent reprograming [30], [31], the apoptotic microenvironment could similarly reprogram the neighboring resident and recruited macrophages as a consequence of MFG-E8 secretion. Considering the pro-inflammatory mediators that can be produced by apoptotic EC, such as extra-cellular matrix fragments [37], a local dampening molecule could be essential to attenuate the local inflammatory response due to tissue injury. Apoptotic cells could constitute the initial source of MFG-E8 in the early inflammatory response, before production by activated macrophages [34]. The new role we are postulating for MFG-E8 could be important to maintain local tissue homeostasis by cellular death itself, to promote the pro-repair programming of macrophages and ensure the early presence of a potent apoptotic cell-opsonizing molecule [34]. This would endow MFG-E8 with another potentially crucial function. Indeed, MFG-E8 is critical for apoptotic cell phagocytosis [34] and in macrophage biology [35], [36]. During apoptotic cell engulfment, MFG-E8 opsonizes phosphatidylserine, allowing its recognition by macrophages through α_v_β_3_ and α_v_β_5_ integrins [34]. This process seems to occur in activated macrophages through a granulocyte-macrophage-colony-stimulating-factor induced mechanism of MFG-E8 expression [35]. In response to bacterial lipopolysaccharides (LPS) stimulation, MFG-E8 has been shown to reduce macrophage activation by modulating integrin signaling [36]. Data from an ischemia-reperfusion injury model indicates that MFG-E8 administration protects mice by promoting apoptotic cell engulfment [44]. MFG-E8 may bind lung collagen to facilitate its clearance in pulmonary fibrosis [45]. The role of MFG-E8 in inflammation extends beyond phagocytosis. Effectively, local release of TGF-β_1_ and CCL22 through MFG-E8 expression may foster the recruitment and maintenance of FoxP3+ Tregs, promoting allograft tolerance [35]. MFG-E8 can modify macrophage behavior by increasing IL-10 production [36]. Significantly, most of these studies have implicated macrophages as the main source of MFG-E8 production and studied its role as an inhibitor of LPS stimulation. We suggest here that apoptotic cell-conditioned media and MFG-E8 reprogram macrophages through increased STAT-3 phosphorylation. However, the signaling pathways involved in STAT-3 activation by MFG-E8 during SS are still unknown. After LPS treatment, MFG-E8 could induce STAT-3 and suppressor of cytokine signaling-3 (SOCS3) activation to attenuate the pro-inflammatory stimulation of macrophages [46]. STAT-3 has recently been implicated in MFG-E8 stimulation of cancer stem cells produced by tumor-associated macrophages [47].

In clinical situations, such as transplant vasculopathy and highly proliferative cancers, where EC apoptosis is important, the constant presence of apoptotic EC could promote an unregulated repair response by macrophages with the constant production of pro-fibrotic and immunosuppressive mediators. This could lead to tissue fibrosis and/or impaired immune response. Therefore, better understanding of MFG-E8's role in macrophage reprogramming and associated signaling pathway activated by the apoptotic cell microenvironment, is central to the development of new therapeutic approaches in transplantation and cancer biology.

## Materials and Methods

### Cell culture and generation of conditioned media

Human umbilical vein endothelial cells (HUVECs) (Clonetics, San Diego, CA, USA) were cultured as described elsewhere [37] and used at passages 4–5. Serum-free media conditioned by apoptotic or caspase-inhibited EC, were obtained as described (42). Equal EC numbers (2.5×10^4^ cells/cm2) were preincubated for 2 h in normal medium containing either DMSO (vehicle) or DEVD-FMK (100 µM) (R&D Systems, Minneapolis, MN, USA) for caspase-3 inhibition, washed and the culture medium was changed for serum-free RPMI medium (Wisent, St-Bruno, Québec, Canada) and then EC were serum-starved for 4 h to obtain SSC-DMSO and SSC-DEVD respectively. To induce apoptosis in another way, EC were treated with MMC (0.01 mg/ml, Sigma, Oakville, Ontario, Canada) for 15 h. Conditioned media were collected and stored at −20°C. Conditioned media were centrifuged at 50 kG to eliminate apoptotic bodies. Necrotic conditioned media were produced by submitting EC to 3 freeze-thaw cycles. Sequential centrifugation protocol was done with 30 mL of SSC with proteases inhibitors (PMSF, Pepstatine A 2 mM, Leupeptine 2 mM), 10 mL of total SSC unfractioned was kept. 20 mL of this total SSC was then centrifuged at 50 000 g at 4°C for 15 min, blebs pellets was then resuspend in 20 mL of SS supplemented of proteases inhibitors and 10 mL were kept aside. The residual 10 mL were then ultracentrifuged at 200 000 g at 4°C for 18 h. Supernatants were kept and small membrane vesicle pellets were resuspended in 10 mL of SS supplemented with proteases inhibitors.

### Murine endothelial cell (MEC) isolation

Thoracic aortae were removed surgically from anaesthetized mice, the endothelial side was placed on Matrigel (BD Bioscience, Mississauga, Ontario, Canada) for approximately a week, or until endothelial spreading was sufficient, in Dulbecco's modified Eagle's medium (DMEM) low glucose (Gibco, Burlington, Ontario, Canada) supplemented with fetal bovine serum (FBS)(Wisent, St-Bruno, Québec, Canada), calf serum (Gibco, Burlington, Ontario, Canada), endothelial cell growth supplement (VWR, Radnor, Pennsylvania, USA), heparine (Sigma, Oakville, Ontario, Canada), fugizon (Gibco, Burlington, Ontario, Canada) and penicillin/streptomycin (Wisent, St-Bruno, Québec, Canada). When the desired confluence was reached, MEC were harvested after dispase treatment and seeded on plastic flasks(BD Bioscience, Mississauga, Ontario, Canada). Cultured MEC were expanded between passages 4 and 6 and tested experimentally.

### Isolation of blood monocyte-derived macrophages and preparation of bone marrow-derived macrophages

Peripheral monocytes were isolated from healthy donors by Ficoll gradient (Wisent, St-Bruno) followed by CD14+ immunomagnetic selection (Stem Cell, Vancouver). Both CD14++/CD16− and CD14low/CD16+ monocyte populations were collected. HMDM were matured in Iscove DMEM (Gibco, Burlington, Ontario, Canada) with penicillin/streptomycin (Wisent, St-Bruno, Québec, Canada), glutamine (Wisent, St-Bruno, Québec, Canada) and 10% decomplemented autologous human serum for 5–7 days before being exposed to experimental media for 24 h. Informed written consent was obtained from healthy donors according to the hospital ethics committee (comité d'éthique de la recherche du CHUM). The data were not shown. Bone marrow-derived macrophages (BMDM) were prepared from C57BL/6 mice. Bone marrow was isolated from femurs by standard sterile techniques and matured for 7 days in culture plastic in Dulbecco's modified Eagle's medium (DMEM) (Wisent, St-Bruno, Québec, Canada) with 10% FBS (Wisent, St-Bruno, Québec, Canada), penicillin/streptomycin (100 µg/ml) (Wisent, St-Bruno, Québec, Canada), and 20% L929 cell-conditioned medium as a source of macrophage-colony stimulating factor. BMDM were more than 96% positive for the macrophage marker F4/80 by flow cytometry.

### Immunoblotting and reagents

In all experiments, equal volumes of all conditioned media were concentrated by centrifugation in a 10-kD vivaspin concentrator, according to the manufacturer's specifications (Sigma, Oakville, Ontario, Canada) as described previously [37] or by a trichloroacetic acid (TCA) precipitation protocol of supernatants 9∶1, washed with cold acetone and solubilization in sample buffer [15]. A fixed volume in all conditions was loaded onto gel. Proteins were separated by SDS-PAGE electrophoresis and transferred to nitrocellulose membranes. Western blotting was performed [37] and the membranes probed with anti-human MFG-E8 and anti-mouse MFG-E8 antibodies (R&D Systems, Minneapolis, MN, USA and Santa Cruz Biotechnology, Santa Cruz, CA, USA, respectively). Ponceau red staining of membranes served as protein-loading control. Proteins were extracted from cell pellets with protease or phosphatase inhibitor cocktail separated by electrophoresis, transferred to nitrocellulose or PVDF membranes, and probed. The membranes were probed with anti-active caspase-3 (Cell Signaling Technology, Pickering, Ontario, Canada), anti-β actin (Abcam, Cambridge, MA, USA), anti-phospho STAT3 and STAT3 total antibodies (Cell Signaling Technology, Pickering, Ontario, Canada).

### Fluorescence microscopy for quantification of cells with chromatin condensation and cell membrane permeabilization

For fluorescence microscopy, unfixed/unpermeabilized adherent EC were stained with Hoechst 33342 (2′-(4-ethoxyphenyl)-5-(4-methyl-1-piperazinyl)-2.5′-bi-1H-benzimidazole) (HO) and PI. They were grown to confluence in 24-well culture plates (BD Bioscience, Mississauga, Ontario, Canada). HO (1 µg/ml) was added to a final concentration of 5 µg/ml immediately before fluorescence microscopy analysis (excitation filter I = 360–425 nm). Apoptotic cells show increased HO fluorescence in the absence of PI positivity. Secondary and primary necrotic cells present PI positivity.

### Enzyme-linked immunosorbent assay (ELISA)

Human IL-6, -8, active TGF-β_1_, MCP-1 protein levels, as well as murine IL-10, MCP-1, macrophage inflammatory protein-2 (MIP-2) and vascular endothelial growth factor (VEGF) were measured by ELISA) according to the supplier's protocol (BD Bioscience, Mississauga, Ontario, Canada and R&D Systems, Minneapolis, MN, USA).

### MFG-E8 immunoprecipitation from MEC and HUVEC SSC

SSC were incubated with specific antibodies against MFG-E8 or control antibody for 6 h. Protein A/G linked beads were incubated overnight at 4°C under agitation. SSC were then centrifuged and immunoblotted for MFG-E8 (Santa Cruz Biotechnology, Santa Cruz, CA, USA).

### Recombinant murine MFG-E8

BMDM matured for 7 days were stimulated with rmMFG-E8 (R&D Systems, Minneapolis, MN, USA) 1 ng/mL in serum-free media (Wisent, St-Bruno, Québec, Canada) for 48 h. PBS added to serum-free media (Wisent, St-Bruno, Québec, Canada) served as negative control. Cytokines/chemokines from the supernatants were evaluated by ELISA.

### Experimental animals and induction of experimental peritonitis

C57BL/6 and BALB/c mice were bought from Charles River (Canada). We acquired the MFG-E8 KO mice on the C57BL/6 background were generously donated to us by Professor S. Nagata. Caspase-3 KO mice were obtained from Jackson Laboratory. Mice were housed in CRCHUM animal facilities. They were injected intraperitoneally (IP) with 0.5 mL of conditioned media or rmMFG-E8 (0.6 µg) for 3 h and then injected with 3% Brewer's thioglycollate (BTG) (Difco) and underwent peritoneal lavage with 5 mL of phosphate buffered saline (PBS) (Wisent, St-Bruno, Québec, Canada) at 2 h thereafter. Peritoneal lavage fluid was centrifuged and stored at −80°C until analyzed for cytokine/chemokine production by ELISA. Macrophages were immunomagnetically-isolated with magnetic beads (Miltenyi Biotech, Auburn, CA, USA) after the peritoneal lavages and kept for immunoblotting. Performed animal experiments were approved by our institutional Animal Care Committee (Comité institutionnel de protection des animaux, CRCHUM).

### Statistical analysis

The results are expressed as mean ± SD were analyzed by Student's *T*-test (with Bonferroni correction when appropriate) or ANOVA, as appropriate. *p*<0.05 was deemed to be significant for all tests.
